# A novel read-through transcript JMJD7-PLA2G4B regulates head and neck squamous cell carcinoma cell proliferation and survival

**DOI:** 10.18632/oncotarget.14081

**Published:** 2016-12-21

**Authors:** Yingduan Cheng, Yi Wang, Jiong Li, Insoon Chang, Cun-Yu Wang

**Affiliations:** ^1^ The Division of Oral Biology and Medicine, School of Dentistry, UCLA, Los Angeles, California, USA

**Keywords:** head and neck squamous cell carcinoma, conjoined gene, JMJD7-PLA2G4B, cell cycle, apoptosis

## Abstract

Recent findings on the existence of oncogenic fusion genes in a wide array of solid tumors, including head and neck squamous cell carcinoma (HNSCC), suggests that fusion genes have become attractive targets for cancer diagnosis and treatment. In this study, we showed for the first time that a read-through fusion gene JMJD7-PLA2G4B is presented in HNSCC, splicing neighboring jumonji domain containing 7 (JMJD7) and phospholipase A2, group IVB (PLA2G4B) genes together. Ablation of JMJD7-PLA2G4B significantly inhibited proliferation of HNSCC cells by promoting G1 cell cycle arrest and increased starvation-induced cell death compared to JMJD7-only knockdown HNSCC cells. Mechanistically, we found that JMJD7-PLA2G4B modulates phosphorylation of Protein Kinase B (AKT) to promote HNSCC cell survival. Moreover, JMJD7-PLA2G4B also regulated an E3 ligase S-phase kinase-associated protein 2 (SKP2) to control the cell cycle progression from G1 phase to S phase by inhibiting Cyclin-dependent kinase inhibitor 1 (p21) and 1B (p27) expression. Our study provides novel insights into the oncogenic control of JMJD7-PLA2G4B in HNSCC cell proliferation and survival, and suggests that JMJD7-PLA2G4B may serve as an important therapeutic target and prognostic marker for HNSCC development and progression.

## INTRODUCTION

Read-through transcripts (also known as conjoined genes) are fusion genes resulting from a hybridization of juxtaposed genes from the same chromosome in the same orientation [1–2]. Fusion genes were first recognized in hematologic malignancies as essential drivers of development and progression of cancer [3–4]. Since then, the total number of gene fusions reported in numerous cancers is estimated to be 10,000, where more than 90% of the discoveries occurred in the past 5 years due to recent bioinformatics advancements [4,5]. Studies report that gene fusions occur in all malignancies and account for about 20% of malignant solid tumor morbidity [1]. Thyroid carcinoma was the first epithelial tumor type found to involve a gene fusion, RET-CCDC6, in carcinoma pathogenesis [6, 7]. Additionally, a high frequency of gene fusions was detected in prostate [8], lung [9], and breast cancer [10], suggesting recurrent fusion genes may serve as valuable biomarkers and drug targets in epithelial cancers. However, the relative mechanism by which fusion genes promote the progression of cancer remains largely unknown.

Squamous cell carcinoma (SCC) is a common and deadly malignancy associated with an uncontrolled growth of cells originating from the mucosal lining of the oral cavity, head and neck, lung, cervix, skin, etc [11, 12]. SCC comprises 90% of head and neck malignancies and is associated with a poor prognosis and the lowest 5-year survival rate amongst major cancers [12]. Recently, a number of studies reported the important role of fusion genes that result from genomic rearrangements and translocations in HNSCC development and metastasis [13, 14]. The MYB-NFIB fusion gene resulting from chromosome translocation in carcinoma of the breast and head and neck has been shown to be oncogenic and a potential therapeutic target [13]. Fibroblast growth factor receptor 3-transforming acidic coiled-coil-containing protein 3 (FGFR3-TACC3) fusion transcript is recurrently detected in HNSCC and has been shown to play a key role in tumor resistance [14]. However, fusion genes still remain poorly understood and, in particular, read-through transcripts have not yet been characterized in HNSCC. Clearly, there is an urgent need for understanding the functional role and molecular mechanisms of these potent oncogenic fusions genes in HNSCC tumorigenesis and progression.

Previously, we found that JMJD7 siRNA was able to inhibit the invasive growth of human SCC23 cell lines in our functional screening assays in vitro [15]. JMJD7 is a JmjC-only group member of the JmjC-domain-containing histone demethylation proteins [16]. JMJD7 was recently identified to affect prostatic cancer DU145 cell viability and found to be highly expressed in SCC [17]. Interestingly, we found that JMJD7 gene is located on chromosome 15 and may form a naturally occurring read-through transcript with a neighboring PLA2G4B gene to encode a fusion protein known as JMJD7-PLA2G4B [18]. JMJD7-PLA2G4B shares an amino acid sequence, including a partial JmjC domain and downstream C2 and phospholipase A2 domains [18]. JMJD-PLA2G4B is a paralog gene of PLA2G4B and has been described previously in U937 cells, isolated from the histiocytic lymphoma, to encode a calcium-dependent phospholipase that hydrolyzes the phospholipids to lysophospholipids and fatty acids [18, 19]. In addition, the human cancer tissue proteome revealed that JMJD7-PLA2G4B proteins are highly expressed in various cancers such as breast, prostate, and thyroid cancer [20]. However, the functional role of JMJD7-PLA2G4B gene in HNSCC and other solid tumors is unknown. In this study, we sought to characterize the expression of JMJD7-PLA2G4B gene in SCC cell lines from diverse head and neck tumor regions, and explore the oncogenic role of JMJD7-PLA2G4B in HNSCC.

## RESULTS

### JMJD7-PLA2G4B is expressed in human HNSCC cell lines

**Figure 1:**
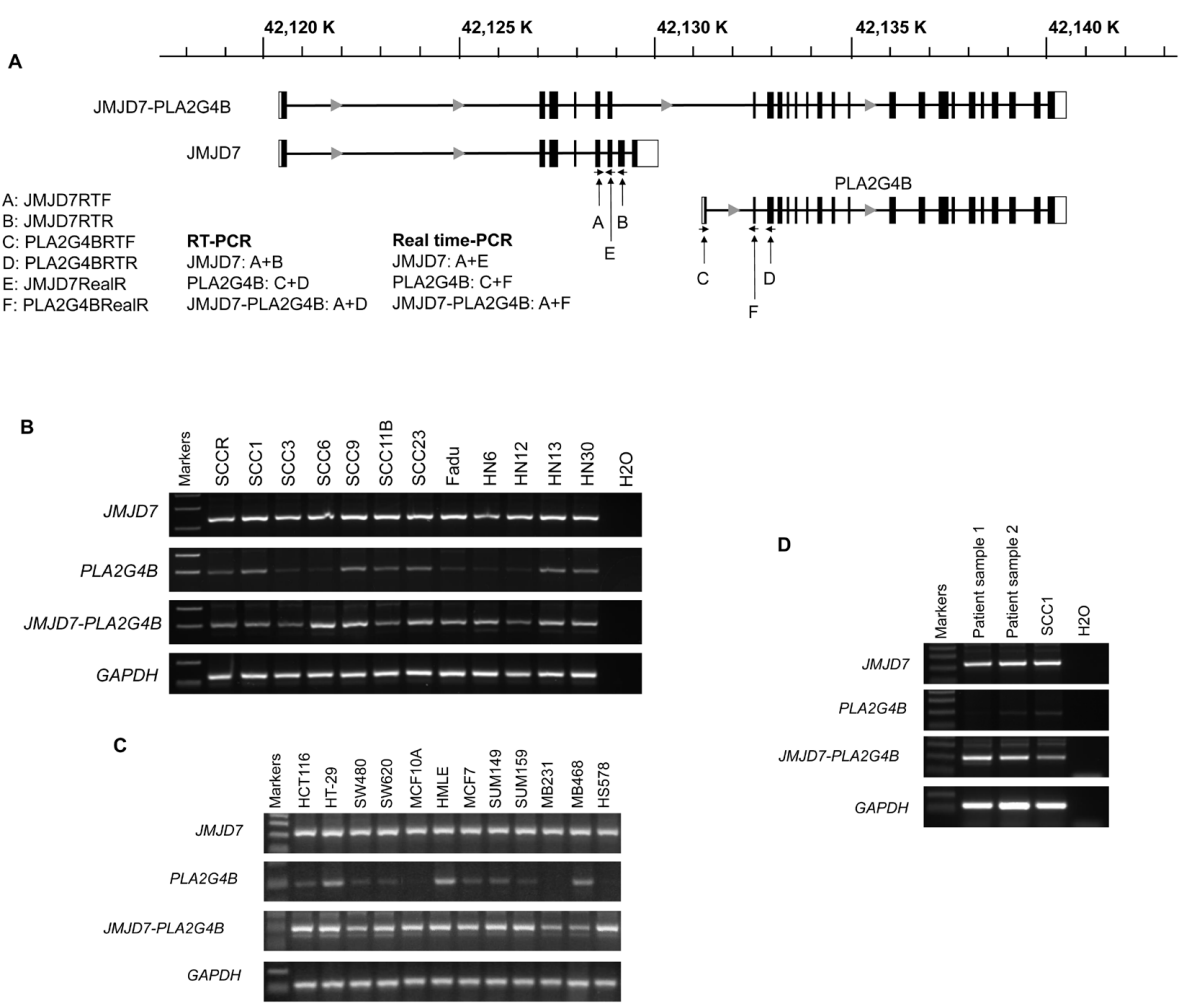
JMJD7-PLA2G4B expression in HNSCC cells **A.** Schematic presentation of gene structure of JMJD7, PLA2G4B, and read-through transcript JMJD7-PLA2G4B and location of the primer design site for each gene products, respectively. **B.** RT-PCR result of JMJD7, PLA2G4B, and JMJD7-PLA2G4B in HNSCC cell lines. **C.** RT-PCR result of JMJD7, PLA2G4B, and JMJD7-PLA2G4B in human colon and breast cancer cell lines and tissues. The representative results from three independent experiments are shown. **D.** RT-PCR result of JMJD7, PLA2G4B, and JMJD7-PLA2G4B in primary HNSCC tissues from patients.

We first investigated the existence of the read-through fusion gene JMJD7-PLA2G4B in human HNSCC cell lines and other solid tumor cell lines. The primers for reverse transcription RT-PCR (RT-PCR) were designed to amplify human JMJD7, PLA2G4B, and JMJD7-PLA2G4B genes according to cDNA sequences from NCBI GenBank (Figure 1A). RT-PCR showed that both JMJD7 and JMJD7-PLA2G4B genes were ubiquitously expressed in all HNSCC cell lines that were examined while PLA2G4B was only detected in few HNSCC cell lines (Figure 1B). JMJD7 and JMJD7-PLA2G4B genes were also expressed in various human colon and breast cancer cell lines (Figure 1C). Importantly, JMJD7-PLA2G4B was detected in primary HNSCC tissues from patients (Figure 1D). This conserved expression of both JMJD7 and JMJD7-PLA2G4B gene amongst various cancer cells and tissues may indicate their important role in oncogenesis.

### JMJD7-PLA2G4B is required for HNSCC proliferation

**Figure 2:**
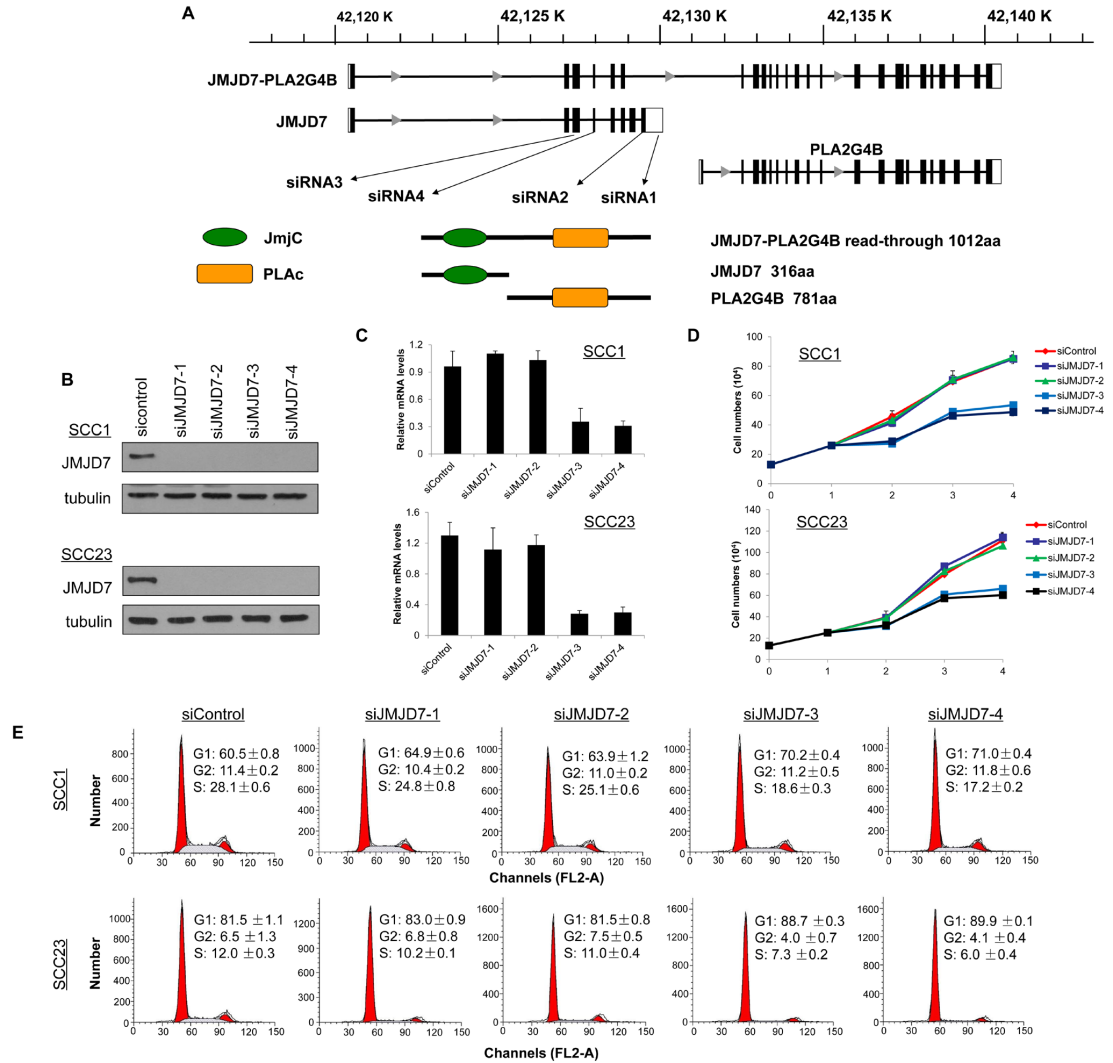
JMJD7-PLA2G4B is required for HNSCC cell proliferation and cell cycle progression **A.** Schematic presentation of gene structure of JMJD7, PLA2G4B, and JMJD7-PLA2G4B and the protein domain structure of JMJD7, PLA2G4B, and JMJD7-PLA2G4B and the size of the product. The locations of regions where siRNAs were designed from are indicated. **B.** Western blotting of JMJD7 in SCC1 and SCC23 cells following transfection with siRNAs. α-tubulin was used as a loading control. **C.** qRT-PCR showing the mRNA level of JMJD7-PLA2G4B after siRNA transfection. Values are means ± S.D; ** p < 0.01. **D.** Knockdown of JMJD7-PLA2G4B using siJMJD-3 and siJMJD-4 decreased the proliferation rate of SCC1 and SCC23 compared to JMJD7 knodckdown (siJMJD7-1 and siJMJD7-2) alone and control. Values are means ± S.D. **E.** Flow cytometry analysis of SCC1 and SCC23 cells after JMJD7 or JMJD7-PLA2G4B knockdown using siRNAs.

We next examined the role of JMJD7 and JMJD7-PLA2G4B in SCC1 and SCC23 cell lines that were previously studied for JMJD7 function in invasive growth of SCC [18]. To explore the functionality of JMJD7-PLA2G4B, we knocked down JMJD7-PLA2G4B using small interfering RNA (siRNA). The schematic diagram indicates the regions where the siRNAs were designed from the cDNA sequence of JMJD7 and JMJD7-PLA2G4B (Figure 2A). siRNA1 (siJMJD7-1) and siRNA2 (siJMJD7-2) were designed to target the sequence that only presented on JMJD7 whereas siRNA3 (siJMJD7-3) and siRNA4 (siJMJD7-4) were designed to target the sequence that controls read-through JMJD7-PLA2G4B fusion gene expression. The depletion of JMJD7 and JMJD7-PLA2G4B expression was confirmed in SCC1 and SCC23 cells by western blot analysis and qRT-PCR, respectively (Figure 2B and 2C). We found that JMJD7-PLA2G4B knockdown by siJMJD7-3 and siJMJD7-4 significantly reduced cell proliferation rates in both SCC1 and SCC23 cells over 72 hours in vitro; however, JMJD7-only knockdown by siJMJD7-1 and siJMJD7-2 had no effect on cell proliferation (Figure 2D). Since one of the hallmarks of cancer is the alteration of the cell cycle [21], we also examined the role of JMJD7-PLA2G4B in the HNSCC cell cycle. Flow cytometric analysis of cell cycle with propidium idodide (pI) demonstrated that G1 arrested state in the SCC1 and SCC23 cells was significantly increased in cells knocked down with JMJD7-PLA2G4B (siJMJD7-3 and siJMJD7-4) compared to the control and JMJD7-only knockdown (Figure 2E). Thus, our data suggest that JMJD7-PLA2G4B promotes HNSCC cells proliferation by inhibiting cell cycle arrest in G1 phase.

### JMJD7-PLA2G4B controls AKT phosphorylation to promote SCC cell survival

**Figure 3:**
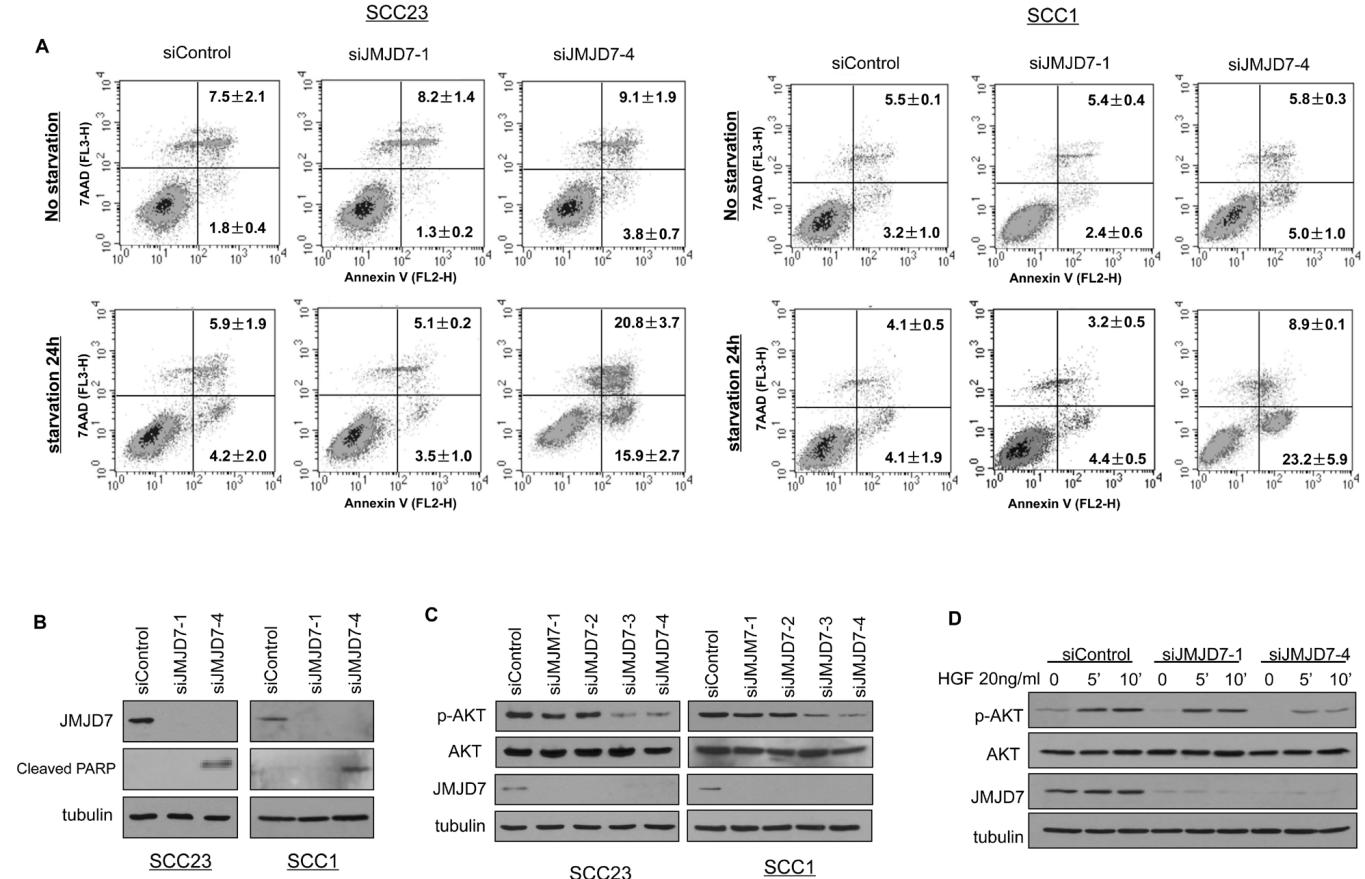
JMJD7-PlA2G4B promotes cell survival and AKT activation in HNSCC cells **A.** Apoptosis detection with Annexin V and pI using flow cytometry for control, JMJD7-only, or JMJD7-PLA2G4B KD SCC23 and SCC1 cells. Cells were serum-starved 24 hours prior to the detection to elicit cell death. **B.** Western blotting of cleaved PARP in SCC23 and SCC1 cells following control, siJMJD7-1, or siJMJD7-4 transfection. **C.** Western blotting of phosphorylated AKT (p-AKT), AKT, and JMJD7 in SCC1 cells following control, siJMJD7-1, siJMJD7-2, siJMJD7-3, or siJMJD7-4 transfection. **D.** Western blotting of HGF-induced AKT phosphorylation in SCC1 cells following control, siJMJD7-1, or siJMJD7-4 transfection.

**Figure 4:**
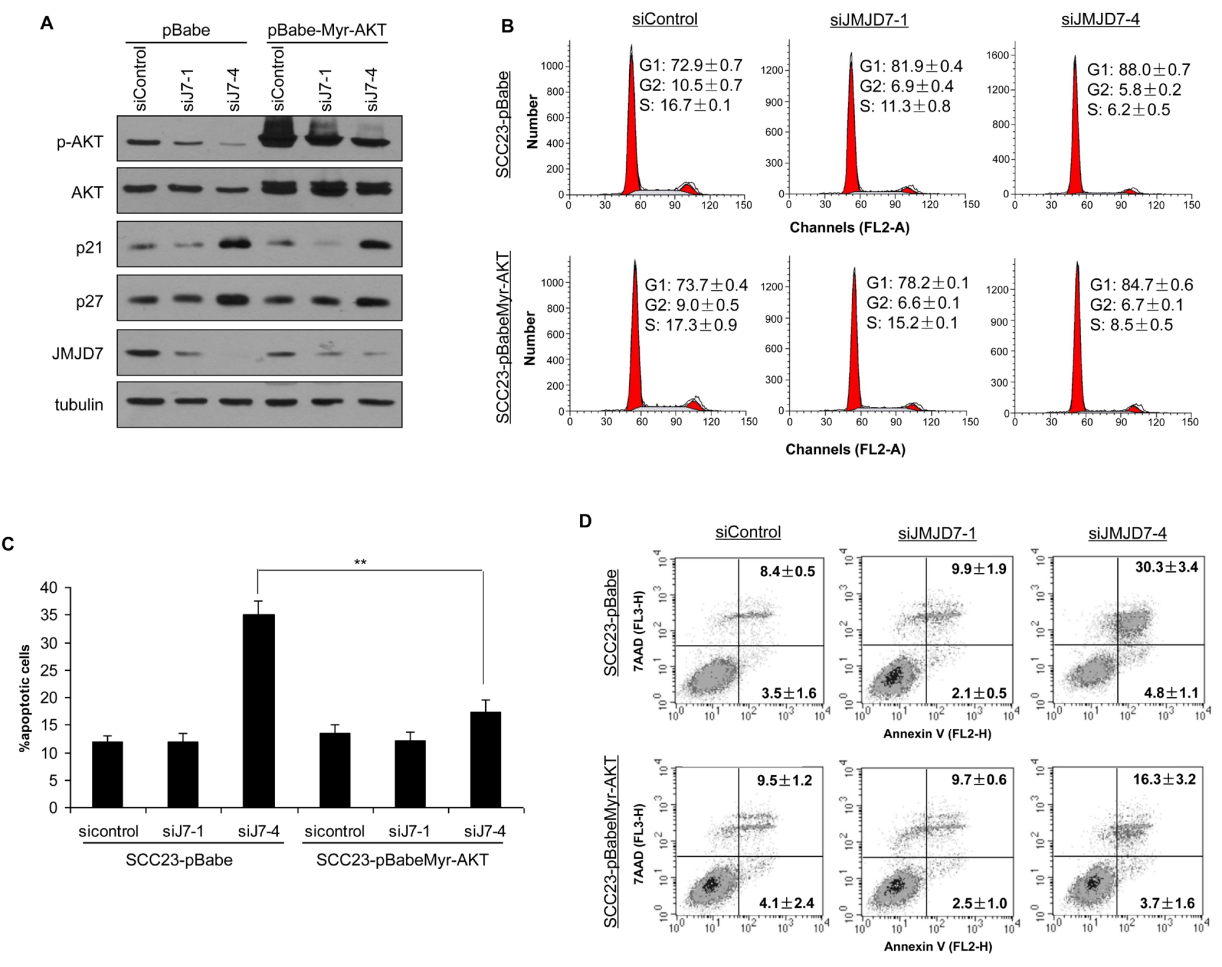
Overexpression of Myr-AKT restores survival in JMJD7-PLA2G4B knockdown cells **A.** Western blotting of p-AKT, AKT, p27, p21, JMJD7 in SCC23 cells knocked down with siJMJD7-1 or siJMJD7-4 first then infected with retroviruses overexpressing Myr-AKT. **B.** Flow cytometry analysis of JMJD7 or JMJD7-PLA2G4B KD SCC23 cells overexpressing Myr-AKT. **C.** Overexpression of Myr-AKT reduced apoptosis in JMJD7-PLA2G4B knockdown SCC23 cells. **D.** Annexin V analysis of SCC23 cells expressing Myr-AKT after JMJD7 or JMJD7-PLA2G4B knockdown.

Using the Annexin V Apoptosis assay, we found that JMJD7-PLA2G4B knockdown increased starvation-induced cell death by apoptosis in both SCC1 and SCC23 compared to the control and JMJD7-only knockdown cells (Figure 3A). Western blotting also confirmed that JMJD7-PLA2G4B knockdown promoted the cleavage of poly ADP ribose polymerase (PARP) (Figure 3B), an indicator of apoptosis in both cell lines [22]. Additionally, JMJD7-PLA2G4B knockdown dramatically inhibited AKT phosphorylation, a major mediator of cell survival and growth in many cancer cells [23], in both SCC1 and SCC23 cell lines (Figure 3C). Furthermore, blocking JMJD7-PLA2G4B in SCC1 cells also inhibited AKT phosphorylation which was induced by hepatocyte growth factor (HGF), a growth factor that was previously shown to activate AKT in HNSCC cells [24] (Figure 3D). To further confirm that AKT activation is required for JMJD7-PLA2G4B-mediated HNSCC cells proliferation and survival, we overexpressed the active myristalated form of AKT (Myr-AKT) in SCC23 cells knocked down with JMJD7-only or JMJD7-PLA2G4B to restore the JMJD7-PLA2G4B loss of function (Figure 4A). Interestingly, overexpression of active AKT had no effect on Cyclin-dependent kinase (CDK) inhibitor p21 and p27 expression in both JMJD7-only and JMJD7-PLA2G4B knockdown cells (Figure 4A) and cell cycle progression (Figure 4B). However, overexpression of active AKT significantly rescued the serum-starvation induced cell death (Figure 4C and 4D) in JMJD7-PLA2G4B knockdown cells compared to control and JMJD7-only knockdown cells.

### JMJD7-PLA2G4B regulates expression of various cell cycle associated oncogenes including SKP2 in HNSCC

**Figure 5:**
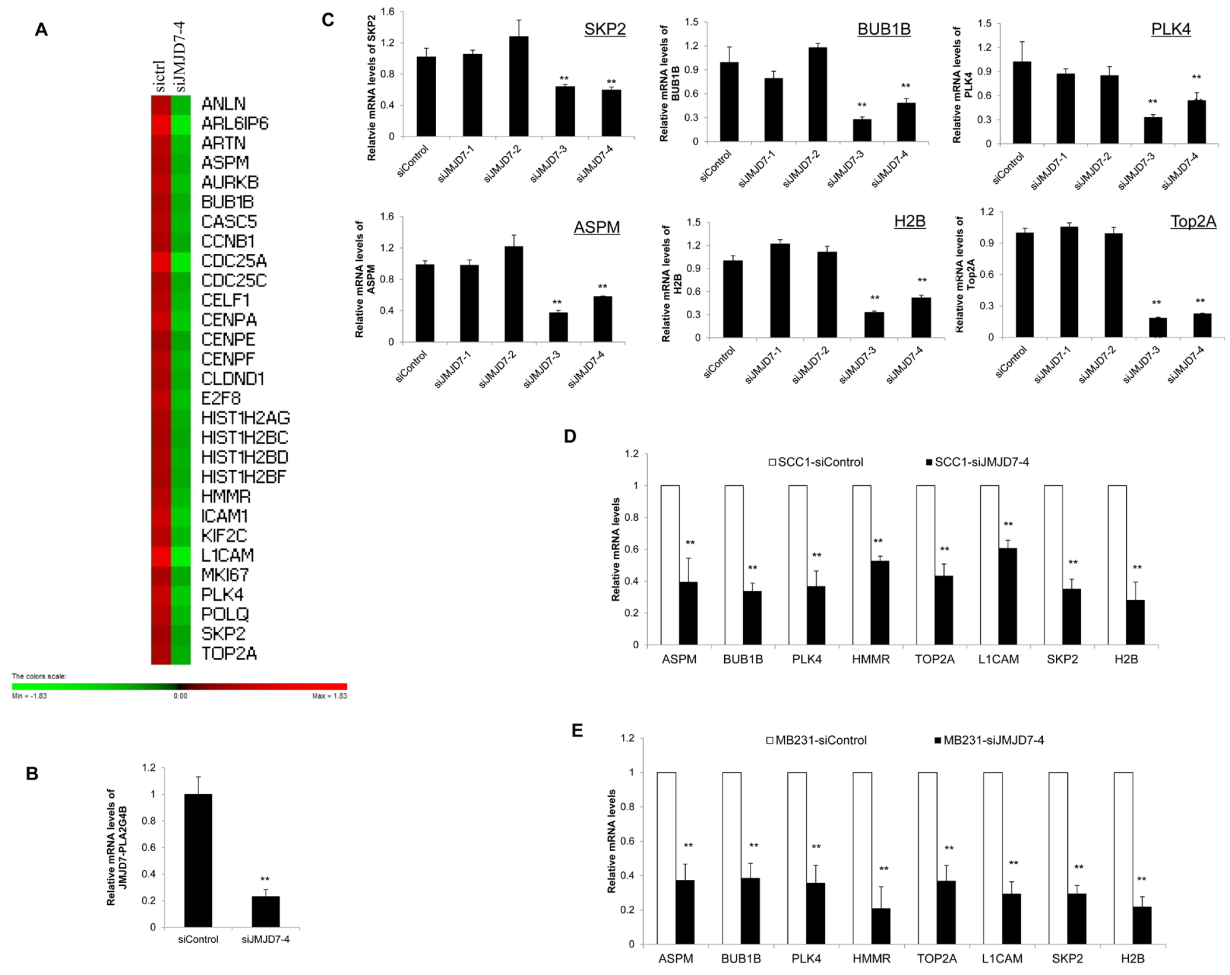
JMJD7-PLA2G4B controls SKP2 expression in HNSCC cells **A.** Gene expression profiling of JMJD7-PLA2G4B target genes in SCC23 cells transduced with scramble (sictr1) or JMJD7-PLA2G4B siRNA (siJMJD7-4). **B.** Real-time RT-PCR showing the mRNA level of JMJD7-PLA2G4B after siRNA transfection with siJMJD7-4. Values are means ± S.D; ** p < 0.01. **C.** Read-time RT-PCR analysis of the expression of SKP2, BUB1B, PLK4, ASPM, H2B, and Top2A in SCC23 cells transduced with control, siJMJD7-1, siJMJD7-2, siJMJD7-3, or siJMJD7-4. Values are means ± S.D; ** p < 0.01. **D.** mRNA expression of SKP2, BUB1B, PLK4, ASPM, H2B, and Top2A in SCC1 cells transduced with control or siJMJD7-4. Values are means ± S.D; ** p < 0.01. E. mRNA expression of SKP2, BUB1B, PLK4, ASPM, H2B, and Top2A in MB231 cells transduced with control or siJMJD7-4. Values are means ± S.D; ** p < 0.01.

To identify potential genes that were regulated by JMJD7-PLA2G4B to promote HNSCC cell proliferation, we compared the gene expression profile between JMJD7-PLA2G4B knockdown (siJMJD7-4) SCC23 cells and control cells using microarray. Gene Set Enrichment Analysis (GSEA) revealed that the knockdown of JMJD7-PLA2G4B significantly reduced the expression of various cell cycle-associated genes (Figure 5A), including S-phase kinase-associated protein 2 (SKP2), Topoisomerase 2-alpha (TOP2A), Polo-like kinase 4 (PLK4), Abnormal spindle-like microcephaly-associated protein (ASPM), BUB1 mitotic checkpoint serine/threonine kinase B (BUB1B), Hyaluronan mediated motility receptor (HMMR), and L1 cell adhesion molecule (L1CAM). Microarray results were confirmed by qRT-PCR showing that JMJD7-PLA2G4B knockdown (Figure 5B) significantly inhibited the expression of these genes in SCC23 cells (Figure 5C) and SCC1 cells (Figure 5D) compared to control and JMJD7-only knockdown cells. Consistently, these genes were also downregulated in JMJD7-PLA2G4B knockdown breast cancer MB231 cells compared to the control (Figure 5E).

### JMJD7-PLA2G4B controls SKP2 expression to regulate cell progression and growth in HNSCC

**Figure 6:**
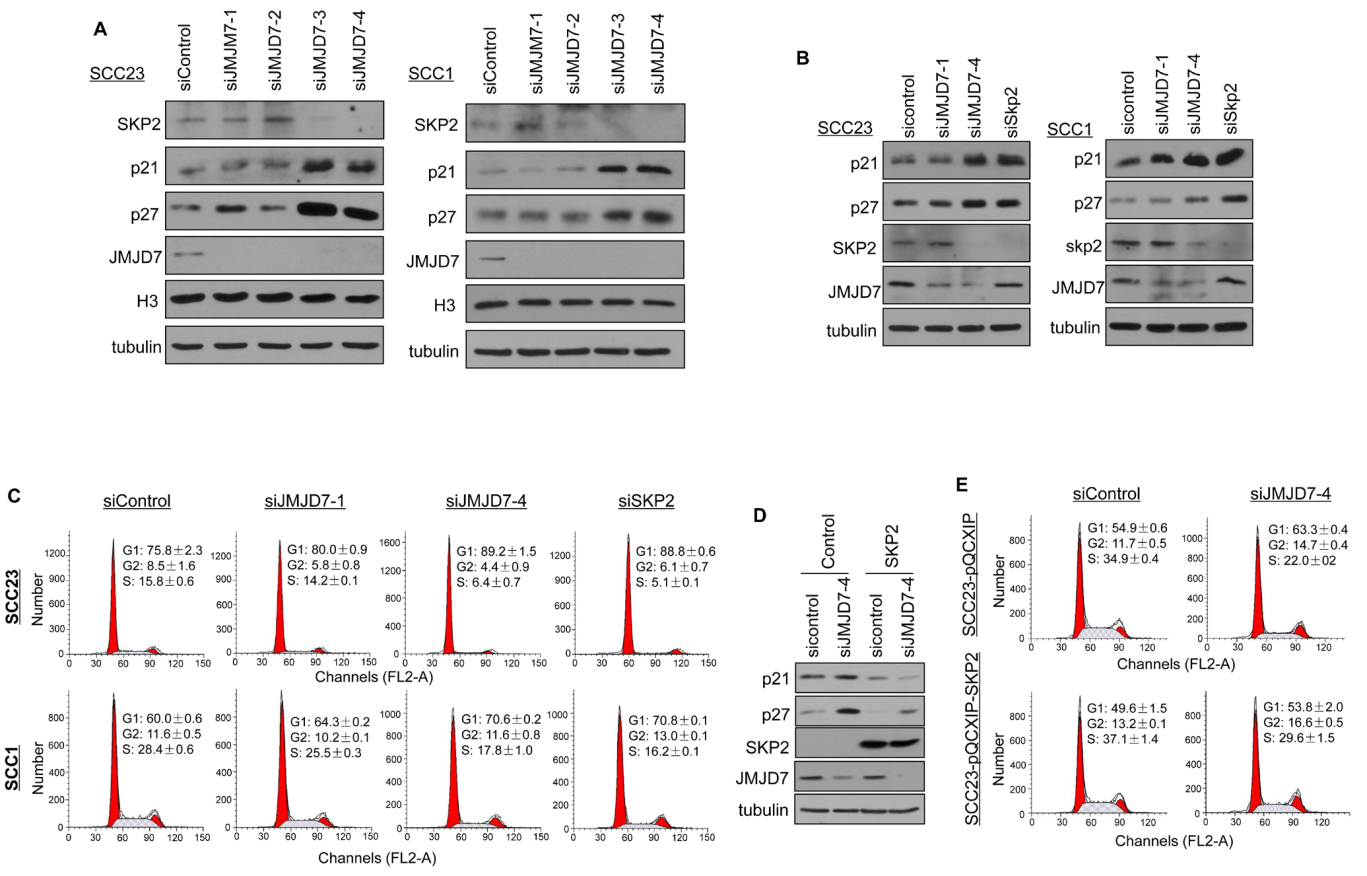
JMJD7-PLA2G4B controls SKP2 expression to promote cell cycle progression in HNSCC cells **A.** Western blotting of SKP2, p21, p27, JMJD7, histone 3 (H3) in SCC23 and SCC1 cells knocked down with control, siJMJD7-1, siJMJD7-2, siJMJD7-3, or siJMJD7-4. **B.** Western blotting of p21, p27, SKP2, and JMJD7 in SCC23 and SCC1 cells knocked down with control, siJMJD7-1, siJMJD7-4, or siSKP2. **C.** Flow cytometry analysis of SCC1 and SCC23 cells after JMJD7, JMJD7-PLA2G4B, or SKP2 knockdown using siRNAs. **D.** Western blotting of p21, p27, SKP2, and JMJD7 in JMJD7-PLA2G4B Knockdown SCC23 cells overexpressing SKP2. **E.** Flow cytometry analysis on JMJD7-PLA2G4B knockdown SCC23 cells overexpressing SKP2.

Amongst the JMJD7-PLA2G4B targeted genes found from the GSEA, we selected SKP2 gene to further examine the association between SKP2 and JMJD7-PLA2G4B in HNSCC. SKP2 is a component of SKP2, Cullin, F-box containing complex (SKP2-SCF) E3 ligase and has previously been reported to inhibit p21 and p27 expression in solid tumors [25, 26]. Western analysis showed that knockdown of JMJD7-PLA2G4B inhibited the SKP2 expression in contrast to the elevated level of p21 and p27 in SCC1 and SCC23 cells (Figure 6A). However, JMJD7-only knockdown had no significant effect on SKP2, p21, and p27 expression in both cell lines. Consistently, knockdown of SKP2 in SCC1 and SCC23 cells mimicked the JMJD7-PLA2G4B knockdown effect by increasing p21 and 27 levels (Figure 6B) and inhibiting G1 arrest (Figure 6C). Furthermore, the restoration of SKP2 in JMJD7-PLA2G4B knockdown SCC23 cells inhibited p21 and p27 expression and G1 arrest, rescuing the cell cycle progression (Figure 6D and 6E). Together, our data suggests that JMJD7-PLA2G4B promotes cell cycle progression by altering SKP2 expression in HNSCC.

## DISCUSSION

In this study, we report for the first time that the read-through fusion gene product JMJD7-PLA2G4B plays a critical role in HNSCC cell proliferation and survival. Our study uncovers the significance of the fusion protein and its role in inappropriate proliferation in HNSCC. We showed that overexpression of JMJD7-PLA2G4B is ubiquitously present in HNSCC, colon, and breast cancer cell lines. Our data also demonstrate that JMJD7-PLA2G4B functions independently from JMJD7 and is essential for HNSCC cell survival, proliferation, and cell cycle progression from G1 arrest.

Based on our findings, JMJD7-PLA2G4B plays a critical role in the regulation of AKT phosphorylation in HNSCC. In HNSCC, HGF can directly activate PI3K or through a crosstalk with the epidermal growth factor receptor (EGFR), leading to AKT activation [24, 27, 28]. Interestingly, our data revealed that knockdown of JMJD7-PLA2G4B inhibits HGF-induced AKT phosphorylation in HNSCC. Thus, further studies are needed to elucidate the role of JMJD7-PLA2G4B on signal transduction leading to AKT activation. The AKT pathway has been known to promote survival and growth of various cancer cells including HNSCC by modulating the downstream apoptotic regulators [27, 29]. AKT activation is also frequently found in human HNSCC tumor tissues and closely correlates with the disease progression [29]. AKT has been previously reported to phosphorylate both p27 and p21 and prevent the nuclear import of p27 as well as the ability of p21 to bind to CDK [30], ultimately enabling cell cycle progression in cancer cells. In contrast, our data showed that while the overexpression of the activated AKT in JMJD7-PLA2G4B knockdown SCC cells was able to restore the ability to resist cell death, it could not reverse G1 phase arrest of the cell cycle. This difference may be context-dependent. Consistent with our data, it has been reported that p21 is not regulated by PI3K-AKT activation in HNSCC cell lines [31], indicating AKT may not be a major cell cycle regulator in HNSCC cells.

Our GSEA data revealed that several cell proliferation and cell-cycle-related oncogenes such as TOP2A, PLK4, ASPM, BUB1B, HMMR, L1CAM, and SKP2 are regulated by JMJD7-PLA2G4B. Specifically, SKP2 is an E3 ligase that has been known to regulate AKT ubiquitination and activation as well as degradation of the CDK inhibitors p21 and p27, which control mammalian cell proliferation [25]. High expression of SKP2 has been implicated in human cancer development and progression including HNSCC and has become a promising therapeutic target [26, 32]. SKP2 also is a useful prognostic marker for HNSCC management because SKP2 is highly expressed in the invasive edge of human HNSCC tumor tissues and associated with lymph node-metastasis cases [26]. Due to its role in AKT activation and p21 and p27 degradation, we examined the role of JMJD7-PLA2G4B on SKP2 regulation in HNSCC cells. We found that JMJD7-PLA2G4B controls cell-cycle progression by regulating SKP2 expression to ultimately modulate the expression of the CDK inhibitors p21 and p27. Thus, based on our data, it is possible that AKT has a more significant effect on cell survival while SKP2 mainly regulates the cell cycle progression by altering p21 and p27 expression in HNSCC. Previously, Gao et al. showed that AKT directly regulates SKP2 by phosphorylation at Serine 72 in cervical and prostate cancer cells [33]. However, because the overexpression of activated AKT did not have effects on the expression of p21 and p27, it was unlikely that JMJD7-PLA2G4B activated AKT to phosphorylate SKP2 at Serine 72 in HNSCC. Thus, based on our data, it is possible that AKT has a more significant effect on cell survival while SKP2 mainly regulates the cell cycle progression by altering p21 and p27 expression in HNSCC. In the future studies, it will be interesting to examine how JMJD7-PLA2G4B regulates the expression of SKP2. Taken together, our findings reveal a novel oncogenic read-through transcript and its functions in HNSCC cell proliferation and survival, implicating JMJD7-PLA2G4B as a potential target for cancer therapy.

## MATERIALS AND METHODS

### Cell culture and reagents

Human HNSCC cell lines (SCCR, SCC1, SCC3, SCC6, SCC9, SCC10PT, SCC11B, SCC23, Fadu, HN6, HN12, HN13, HN30), human colon cancer cell lines (HCT116, HT29, SW480, and SW620), and human breast cancer cell lines (MCF7, MB231, MB468, and HS578) were cultured in Dulbecco's modified Eagle's medium (DMEM) with 10% FBS with 1% penicillin-streptomycin (Invitrogen), and breast cancer cell line (SUM149 and SUM159) was cultured with Ham's F-12 medium with 5μg/ml insulin, 1μg/ml Hydrocortisone, and 10mM HEPES at 37°C with 5% carbon dioxide. HNSCC cell lines were obtained from Dr. Thomas Carey at the University of Michigan, and the other tumor cell lines were purchased from ATCC. Recombinant human HGF was purchased from R&D systems, reconstituted at 50 μg/mL in sterile PBS containing 0.1% bovine serum albumin, and stored at -80°C as a stock.

### Viral transduction and transfection

The human pBabe-Myr-AKT plasmid (#9015) and the human pcDNA3-myc-Skp2 plasmid (#19947) were purchased from Addgene. The pcDNA3-myc-Skp2 plasmid was subcloned into the retroviral vector pQCXIH with SKP2 ORFF/ORFR sequence (Supplemental Table 1). The PCR reactions were performed with AccuPrimeTM Taq polymerase according to the manufacturer's protocol (Invitrogen), then subcloned into pQCXIP using AgeI and EcoRI site. PureLinkTM HiPure plasmid filter purification kit (Invitrogen) was used for midi preparation of the plasmid. To generate stable cell lines, virus production was performed according to the protocol provided by Addgene using HEK-293T viral packaging cells and Lipofectamine 2000 (Invitrogen). 48 hours after the transfection, the media containing viruses were collected and used for infection. Infected cells were incubated for 48 hours and then selected with puromycin (1μg/ml) for at least 1 week. The expression was confirmed by real-time RT-PCR or Western blot analysis. SKP2 siRNA, PLA2G4B siRNA, and scramble siRNA were purchased from Santa Cruz Biotechnology. Each siRNA consists of pools with three to five target-specific 19-25 nt siRNAs designed to knockdown target gene expression. The target siRNA sequence for JMJD7 and JMJD7-PLA2G4B are listed (Supplemental Table 1) and were synthesized by Sigma Aldrich. Cells were transfected with siRNAs using Lipofectamine RNAiMax (Invitrogen). Cells were collected 36 hours after transfection for further analysis.

### RT-PCR of HNSCC cell lines and tissue

The use of consented human head and neck cancer tissue samples for this study was approved by the UCLA Institutional Review Board. The HNSCC tissues from two independent patients were obtained from UCLA Department of Pathology & Laboratory Medicine (TPCL). Total RNA was isolated from cells and tissues using TRIzol reagent (Invitrogen), and cDNA was synthesized with random hexamers (Sigma) using SuperScript III reverse transcriptase (Invitrogen). Semi-quantitative RT-PCR was performed using AccuPrimeTM Taq DNA Polymerase according to the manufacturer's protocol (Invitrogen). qRT-PCR was carried out with iQ SYBR green supermix (Bio-Rad) on an iCycler iQ real-time PCR detection system (Bio-Rad). All the primer sequences for PCR are listed (Supplemental Table 1). All samples were run in triplicates in the same culture plate.

### Microarray

For microarray, total RNA was extracted from SCC23 cells with miRNeasy kit following the manufacturer's instruction (Qiagen) after 72 hours of transfection with scramble siRNA or siJMJD7-4, respectively. 5 µg aliquots of total RNAs were transcribed from each sample to double-stranded complementary DNA (cDNA) using SuperScript II RT (Invitrogen) with an oligo-dT primer and then used to generate single-stranded RNAs. The biotin-labeled RNAs were fragmented and hybridized with an Affymetrix Human Genome U133 Plus 2.0 Array at the UCLA DNA Microarray Facility. The arrays were scanned with the GeneArray scanner (Affymetrix). The robust multichip average (RMA) method was used to normalize the raw data. Heatmap of differential gene expression was generated using PermutMatrix v1.9.3 to reflect fold change relative to median expression of each gene in Log2 ratio [34]. The genes with more than 2-fold changes in expression were considered significant.

### Cell cycle assays and annexin V apoptosis assays

Cell cycle distribution was determined by DNA content using propidium iodide staining. Cells were fixed in pre-cold 70% ethanol and stored at 4°C before analysis. PE Annexin V Apoptosis Detection Kit I was purchased from BD Pharmingen, and the experiments were done according to the manufacture's instruction. Flow cytometric analysis was done by a FACS SCAN flow cytometer (Becton-Dickinson, San Jose, CA) at the flow cytometry center at University of California, Los Angeles. For each sample, 10,000 events were counted and recorded. All samples were performed in triplicates.

### Cell proliferation assay

SCC1 and SCC23 cells were seeded onto a 6-well culture plate with a density of 1.4 × 105 per well. Then cells were transfected with control siRNA, JMJD7-siRNA1, or JMJD7-siRNA4, respectively. The cell numbers were counted at 0, 24, 48, and 72 hours interval following the transfection using Countess II FL Automated Cell Counters (Invitrogen).

### Western blot

Cells were collected and lysed with whole cell lysate buffer from Sigma-Aldrich with 1/100 protease inhibitor cocktail. 40 μg of lysates were separated by 8-15% SDS-PAGE and transferred to a PVDF membrane using Bio-Rad (Hercules, CA) semidry transfer apparatus. The membranes were incubated with blocking solution containing 5% dry-milk in TBS/Tween 20 buffer at room temperature for 1 hour and subsequently probed with primary antibodies at 4˚C overnight. Appropriate secondary antibodies were used and detected using ECL reagents (Pierce, Rockford, IL, USA) on the Kodak BioMax MS film or Bio-Rad ChemiDoc MP System using Image Lab 5.2.1 version. Primary antibodies to p-AKT, AKT, p21, p27, SKP2, HA, and cleaved PARP were purchased from Cell Signaling Technology. Antibodies to JMJD7 were purchased from Abgent. Antibodies to α-Tubulin were purchased from Sigma-Aldrich.

### Statistical analysis

Experiments presented in the figures are representative of at least three independent repetitions. Data were presented as mean ± SD. Statistical analysis performed with SPSS 23.0 were used in data processing for the paired or two-sided t test or ANOVA. P values < 0.05 were considered significant.

## SUPPLEMENTARY MATERIALS FIGURES AND TABLES



## References

[1] Mitelman F, Johansson B, Mertens F (2007). The impact of translocations and gene fusions on cancer causation. Nat Rev Cancer.

[2] Prakash T, Sharma VK, Adati N, Ozawa R, Kumar N, Nishida Y, Fujikake T, Takeda T, Taylor TD (2010). Expression of conjoined genes: another mechanism for gene regulation in eukaryotes. PLoS One.

[3] Watson IR, Takahashi K, Futreal PA, Chin L (2013). Emerging patterns of somatic mutations in cancer. Nat Rev Genet.

[4] Yoshihara K, Wang Q, Torres-Garcia W, Zheng S, Vegesna R, Kim H, Verhaak RGW (2015). The landscape and therapeutic relevance of cancer-associated transcript fusion. Oncogene.

[5] Mertens F, Johansson B, Fioretos T, Mitelman F (2015). The emerging complexity of gene fusions in cancer. Nat Rev Cancer.

[6] Kumar-Sinha C, Kalyana-Sundaram S, Chinnaiyan AM (2015). Landscape of gene fusions in epithelial cancers: seq and ye shall find. Genome Med.

[7] Pierotti MA, Santoro M, Jenkins RB, Sozzi G, Bongarzone I, Grieco M, Monzini N, Miozzo M, Hermann MA, Fusco A (1992). Characterization of an inversion on the long arm of chromosome 10 juxtaposing D10S170 and RET and creating the oncogenic sequence RET/PTC. Proc Natl Acad Sci USA.

[8] Nacu S, Yuan W, Kan Z, Bhatt D, Rivers CS, Stinson J, Peters BA, Modrusan Z, Jung K, Seshagiri S (2011). Deep RNA sequencing analysis of readthrough gene fusions in human prostate adenocarcinoma and reference samples. BMC Med Genomics.

[9] Fernandez-Cuesta L, Sun R, Menon R, Goerge J, Lorenz S, Meza-Zepeda LA, Peifer M, Plenker D, Heuckmann JM, Leenders F, Zander T, Dahmen I, Koker M (2015). Identification of novel fusion genes in lung cancer using breakpoint assembly of transcriptome sequencing data. Genome Biol.

[10] Varley KE, Gertz J, Roberts BS, Davis NS, Bowling KM, Kirby MK, Nesmith AS, Oliver PG, Grizzle WE, Forero A (2014). Recurrent read-through fusion transcripts in breast cancer. Breast Cancer Res Treat.

[11] Torre L, Bray G, Siegel R, Ferlay J, Lortet-Tieulent J, Jemal A (2012). Global cancer statistics, 2012. CA Cancer J Clinic.

[12] Pulte D, Brenner H (2010). Changes in survival in head and neck cancers in the late 20th and early 21st century: A period analysis. Oncologist.

[13] Persson M, Andren Y, Mark J, Horlings HM, Persson F, Stenman G (2009). Recurrent fusion of MYB and NFIB transcription factor genes in carcinomas of the breast and head and neck. Proc Natl Acad Sci USA.

[14] Daly C, Castanaro C, Zhang W, Zhang Q, Wei Y, Ni M, Young TM, Zhang L, Burova E, Thurston G (2016). FGFR3-TACC3 fusion proteins act as naturally occurring drivers of tumor resistance by functionally substituting for EGFR/ERK signaling. Oncogene.

[15] Ding X, Pang H, Li J, Zhong Q, Chen X, Dry S, Wang CY (2014). Epigenetic activation of AP1 promotes squamous cell carcinoma metastasis. Sci Signal.

[16] Klose R, Kallin E, Zhang Y (2006). JmjC-domain-containing proteins and histone demethylation. Nat Rev Genet.

[17] Zhu S, Xu Y, Song M, Chen G, Wang H, Zhao Y, Wang Z, Li F (2016). PRDM16 is associated with evasion of apoptosis by prostatic cancer cells according to RNA interference screening. Mol Med Rep.

[18] Marcon E, Smiley S, Turinsky A, Greenblatt J, Emili A., Wodak S, Greenblatt J (2014.). Networks of histone demethylases and their relevance to the regulation of chromatin structure and dynamics. Systems analysis of chromatin-related protein complexes in cancer.

[19] Song C, Chang XJ, Bean KM, Proia MS, Knopf JL, Kriz RW (1999). Molecular characterization of cytosolic phospholipase A2-beta. J Biol Chem.

[20] Uhlen M, Fagerberg L, Hallstrom BM, Lindskog C, Oksvold P, Mardinoglu A, Sivertsson A, Kampf C, Sjostedt E, Asplund A, Olsson I, Edlund K, Lundberg E (2015). Proteomics. Tissue-based map of the human proteome. Science.

[21] Hanahan D, Weinberg R (2011). Hallmarks of cancer: the next generation. Cell.

[22] D’Amours D, Desnoyers S, D’Silva I, Poirier GG (1999). Poly(ADP-ribosyl)ation reactions in the regulation of nuclear functions. Biochem J.

[23] Martini M, De Santis MC, Braccini L, Gulluni F, Hirsch E (2014). PI3K/AKT signaling pathway and cancer: an updated review. Ann Med.

[24] Zeng Q, Chen S, You Z, Yang F, Carey T, Saims D, Wang CY (2002). Hepatocyte growth factor inhibits anoikis in head and neck squamous cell carcinoma cells by activation of ERK and Akt signaling independent of NFκB. J Biol Chem.

[25] Chan CH, Li CF, Yang WL, Gao Y, Lee SW, Feng Z, Huang HY, Tsai KK, Flores LG, Shao Y, Hazle JD, Yu D, Wei W (2012). The Skp2-SCF E3 ligase regulates Akt ubiquitination, glycolysis, Herceptin sensitivity, and tumorigenesis. Cell.

[26] Carracedo DG, Astudillo A, Rodrigo JP, Suarez C, Gonzalez MV (2008). Skp2, p27kip1 and EGFR assessment in head and neck squamous cell carcinoma: prognostic implications. Oncol Rep.

[27] Moral M, Paramio JM (2008). Akt pathway as a target for therapeutic intervention in HNSCC. Histol Histopathol.

[28] Chau N, Perez-Ordonez B, Zhang K, Pham N, Ho J, Zhang T, Ludkovski O, Wang L, Chen E, Tsao M, Kamel-Reid S, Siu L (2011). The association between EGFR variant III, HPV, p16, c-MET, EGFR gene copy number and response to EGFR inhibitors in patients with recurrent or metastatic squamous cell carcinoma of the head and neck. Head Neck Oncol.

[29] Amornphimoltham P, Sriuranpong V, Patel V, Benavides F, Conti CJ, Sauk J, Sausville EA, Molinolo AA, Gutkind JS (2004). Persistent activation of the Akt pathway in head and neck squamous cell carcinoma: a potential target for UCN-01. Clin Cancer Res.

[30] Abukhdeir A, Park B (2008). p21 and p27. Expert Rev Mol Med.

[31] Llanos S, Garcia-Pedero JM, Morgado-Palacin L, Rodrigo JP, Serrano M (2016). Stabilization of p21 by mTORC1/4E-BP1 predicts clinical outcome of head and neck cancers. Nat Commun.

[32] Golusinski P, Zhi X, Lamperska K, Luczewski L, Schork NJ, Golusinski W, Masternak M (2015). 15 genetic biomarkers in head and neck squamous cell carcinoma. Oral Oncol.

[33] Gao D, Inuzuka H, Tseng A, Chin RY, Toker A, Wei W (2009). Phosphorylation by Akt1 promotes cytoplasmic localization of Skp2 and impairs APCCdh1-mediated Skp2 destruction. Nat Cell Biol.

[34] Caraux G, Pinloche S (2005). PermutMatrix: a graphical environment to arrange gene expression profiles in optimal linear order. Bioinformatics.

