# Supplementary material for: A novel read-through transcript JMJD7-PLA2G4B regulates head and neck squamous cell carcinoma cell proliferation and survival
Source: Oncotarget. 2016 Dec 21;8(2):1972–82. doi: 10.18632/oncotarget.14081 (PMC5341748; doi:10.18632/oncotarget.14081)
Supplement: Supplementary file 1 [file oncotarget-08-1972-s001.pdf]

A Novel Read-through transcript JMJD7-PLA2G4B Regulates HNSCC Proliferation and Survival

Yingduan Cheng, Yi Wang, Jiong Li, Insoon Chang, and Cun-Yu Wang

SUPPLEMENTAL DATA

Supplementary Table 1

**Primer name**	**Sequence 5'- 3'**
GAPDHh+mF	TCATTGACCTCAACTACATG
GAPDHh+mR	TCGCTCCTGGAAGATGGTGAT
JMJD7RTF	AGCTAACTGAAGAGGGCACC
JMJD7RTR	TCGTATTCCATGTCATACCAG
JMJD7RealR	ACTGTAACTAGGGTACCGTG
PLA2G4BRTF	ATCATTCCTGCTCCTGAGGA
PLA2G4BRTR	CTGCTGTTCTTGACCGTGC
PLA2G4BRealR	CTAGGTCCTTAGAGGGTAGG
jmjd7F	AACCTACCAGCTGACTGAAG
jmjd7R	GGGTGTCCATCAGTTGAAAG
pla2g4bF	TTCCTGTGGACTTGGTCTCA
pla2g4bR	GTGGAAGTTCTGATTCCAGAC
ASPMF	CACAGTCCAGTTTGACCTTC
ASPMR	CAAATGGCATCGGGTGTCTG
BUB1BF	TGGCACAAGAATCTGCCTGT
BUB1BF	CAGAGGGTCATTTCCAGTGT
HMMRF	ATCAAGCATGTTGTGAAGTTG
HMMRR	CCTCTTGAAGTTTTGTCTCAC
L1CAMF	GCCATGTCCCATGAGATCC
L1CAMR	AGGCAGAACCACTGACTCC
PLK4F	ATAGAGTGAAACCCTTCTCAG
PLK4R	GAGGTTAGAAAGTGTGAGGTC
TOP2AF	TGCTGGATCCACCAAAGATG
TOP2AR	GTTTCATCCAACTTGTCCTTC
SKP2F	ATTGTCCGCAGGCCTAAGC
SKP2R	AGAGACCTTTAGCAGCTCAG
H2BEF	CATGAACTCCTTCGTCAACG
H2BErealR	ATGGTGGAGCGCTTGTTGTA
SKP2ORFF	CCGGCGGCCGCGCCACCATGCACAGGAAGCACCTCCA
SKP2ORFR	CTAACCGGTTGACTTATCGTCGTCATCCTTGTAATCT AGACAACTGGGCTTTTGCAG
**SiRNA**	**Sequence 5'- 3'**
JMJD7siRNA1	ACACAGGACAUGUUGAAUA
JMJD7siRNA2	UCUGGUAUGACAUGGAAUA
JMJD7siRNA3	ACAAGGACCACUAUGAGAA
JMJD7siRNA4	UGCACAAGGACCACUAUGA
